# Resident and Program Director’s Perceptions of Milestone-Based Feedback in Obstetrics and Gynecology

**DOI:** 10.1177/2382120518774794

**Published:** 2018-05-20

**Authors:** Eduardo Hariton, Pietro Bortoletto, K Lauren Barnes, Anjali J Kaimal, Amy R Stagg

**Affiliations:** 1 The Vincent Center for Reproductive Biology, Department of Obstetrics & Gynecology, Massachusetts General Hospital, Boston, MA, USA; 2 Reproductive Biology, Department of Obstetrics and Gynecology, Brigham and Women’s Hospital, Boston, MA, USA

**Keywords:** Milestones, milestone-based feedback, resident evaluation

## Abstract

**Introduction:**

In July 2014, US residency programs fully implemented the Next Accreditation System including the use of milestone evaluation and reporting. Currently, there has been little investigation into the result of implementation of this new system. Therefore, this study sought to evaluate perceptions of Obstetrics and Gynecology residents and program directors regarding the use of milestone-based feedback and identify areas of deficiency.

**Methods:**

A Web-based survey was sent to US-based Obstetrics and Gynecology residents and program directors regarding milestone-based assessment implementation.

**Results:**

Out of 245 program directors, 84 responded to our survey (34.3% response rate). Of responding program directors, most reported that milestone-based feedback was useful (74.7%), fair (83.0%), and accurate (76.5%); however, they found it administratively burdensome (78.1%). Residents felt that milestone-based feedback was useful (62.7%) and fair (70.0%). About 64.3% of residents and 74.7% of program directors stated that milestone-based feedback is an effective tool to track resident progression; however, a sizable minority of both groups believe that it does not capture surgical aptitude. Qualitative analysis of free response comments was largely negative and highlighted the administrative burden and lack of accuracy of milestone-based feedback.

**Conclution:**

Overall, both Obstetrics and Gynecology program directors and residents report that milestone-based feedback is useful and fair. Issues of administrative burden, timeliness, evaluation of surgical aptitude, and ability to act on assigned milestone levels were identified. Although this study is limited to one specialty, such issues are likely important to all residents, faculty, and program directors who have implemented the Next Accreditation System requirements.

## Introduction

More than a decade ago, the American Board of Medical Specialties (ABMS) established a conceptual framework and language of 6 domains of clinical competency and moved toward outcomes and learner-centered medical education.^1^ Since then, program director organizations, the specialty colleges, and related academic organizations have worked to create an explicit set of milestones to track the progression of their trainees along a specialty-specific trajectory of professional development.^2,3^ Originated by the Accreditation Council for Graduate Medical Education (ACGME) from 2009 to 2013, the Next Accreditation System’s (NAS) stated goals are

to enhance the ability of the peer-review system to prepare physicians for practice in the 21st century, to accelerate the ACGME’s movement toward accreditation on the basis of educational outcomes; and to reduce the burden associated with the current structure and process-based approach.

The stated intent of these changes is to make the Graduate Medical Education (GME) accreditation process fairer, more balanced, less prescriptive, and less burdensome to residents, program directors, and institutions, with the goal of improving resident education as well as patient care.^3^

In July 2014, all residency programs in the United States including Obstetrics and Gynecology implemented the NAS, incorporating the use of milestone evaluation and reporting.^4^ Prior studies of early use of milestone implementation have demonstrated both benefits and obstacles encountered by residency programs. Benefits cited are early recognition of struggling residents and identification of curriculum gaps in residency programs and additional opportunities for feedback to residents. Reported barriers included increased administrative work, difficulty adopting a “developmental progression model” in the context of distinct clinical rotations, and issues with organizing effective clinical competency committees.^5–7^

To our knowledge, there have been no prior studies to assess how the adoption of the milestone reporting has affected Obstetrics and Gynecology residency programs or what perceptions and attitudes residents have toward this system. This is especially important, as the Obstetrics and Gynecology curriculum must evaluate both medical and surgical proficiency, posing the unique challenge of using this feedback system to evaluate trainees on 2 very different competencies.^8^ Our study seeks to evaluate resident and program director perceptions of milestone-based feedback.

## Methods

As we were not able to find previously published questionnaires that evaluated milestone-based feedback, we created 2 distinct surveys for Obstetrics and Gynecology: 1 for program directors and 1 for residents. Surveys were developed by a team of 5 Ob-Gyn residents and a program director. The surveys were trialed by different residents and academic faculty at our institution and revised as needed for content and clarity. The program director survey consisted of 12 questions aimed at assessing their perceptions and attitudes regarding the usefulness, accuracy, and ease of implementation of milestone-based feedback as compared with traditional feedback methods. The resident survey consisted of 11 questions aimed at assessing perceptions and attitudes regarding the usefulness and accuracy of milestone-based feedback. These attributes were chosen because they were felt to be the main barriers to nationwide adoption and implementation of milestone-based feedback and as such, identifying and correcting any inadequacies in one of these characteristics would likely increase adoption. Both surveys had 5-level Likert scale questions as well as a free response section where program directors and residents were invited to provide additional comments. All questions were optional and could be skipped. The surveys were created for distribution by email for data collection via SurveyMonkey (SurveyMonkey Inc., Palo Alto, CA, USA; www.surveymonkey.com).

An email list of all program directors and program coordinators at ACGME-accredited Obstetrics and Gynecology residency programs was created using information available on the ACOG and APGO Web sites.^9,10^ Both surveys were distributed via email on February 3, 2016. Data collection ended April 3, 2016, 2 months after its initiation. After the initial email, program directors and program coordinators were contacted by email twice with a reminder to either complete the survey or forward the survey to residents, respectively. An entry into a raffle to win an Apple iPad was offered as an incentive for completion of the survey for the residents only. Once completed, data were downloaded and analyzed in Microsoft Excel 2008 (Seattle, WA, USA). National demographic data among Obstetrics and Gynecology residency programs were compiled using the program self-reported information on the American Medical Association FREIDA online database.^11^ Proportion analysis was performed in Stata 10.0 (Stata, College Station, TX, USA) to compare program director and resident responses for overlapping survey questions. Free responses were analyzed using qualitative content analysis, in which consensus codes were reached by 3 of the authors through an iterative process with preliminary and secondary coding. From this analysis, themes emerged into which responses could be categorized.

This survey study was reviewed and approved for exemption by the Partners HealthCare Institutional Review Board.

## Results

Of the 261 Obstetrics and Gynecology US residency program directors, we obtained reliable contact information for 245 program directors and could contact 240 residency coordinators to forward our survey to the residents in their program. Out of 245 program directors, 84 responded to our survey (34.3% response rate). A total of 631 residents responded to our survey. An exact response rate for residents could not be calculated due to inability to accurately track how many residents received our survey via the resident coordinators and program directors; however, there were 5005 US Obstetrics and Gynecology residents at the time of the survey indicating that we received data from 12.6% of eligible residents.

Resident respondents were distributed among all 4 postgraduate year (PGY) classes and all 5 ACOG regions, with no class or region representing more than 30% or less than 10% of respondents (Table 1). About 75% of residents attended academic programs. Program size varied among respondents, with 7.9% attending programs with <12 residents, 55.0% programs with 12 to 28 residents, and 37.1% programs with more than 28 residents. About 91% of residents reported that their program uses milestone-based feedback to evaluate residents.

**Table 1.** Resident demographics.

Residency year	PGY-1	159 (25.2)
	PGY-2	177 (28.1)
	PGY-3	152 (24.1)
	PGY-4	143 (22.7)
Type of program	Academic	473 (75.0)
	Community	156 (24.7)
	Military	2 (0.3)
Program size	<12 total residents	50 (7.9)
	12-28 total residents	347 (55.0)
	>28 total residents	234 (37.1)
Program region	Region 1 (Connecticut, Maine, Massachusetts, Newfoundland, New Hampshire, New York, Rhode Island, Vermont	153 (24.2)
	Region 2 (Delaware, Indiana, Kentucky, Michigan, New Jersey, Ohio, Pennsylvania)	147 (23.3)
	Region 3 (District of Columbia, Florida, Georgia, Maryland, North Carolina, South Carolina, Virginia, West Virginia)	110 (17.4)
	Region 4 (Alabama, Arkansas, Illinois, Iowa, Kansas, Louisiana, Manitoba, Minnesota, Mississippi, Missouri, Nebraska, Oklahoma, Tennessee, Texas, Wisconsin)	111 (17.6)
	Region 5 (Arizona, Armed Forces District, British Columbia, California, Colorado, Hawaii, Nevada, New Mexico, Oregon, Utah, Washington)	110 (17.4)
Program uses milestones	Yes	575 (91.1)
	No	17 (2.7)
	Unsure	39 (6.2)

n = 631. Data are expressed as No. (%).

Program director respondents represented all 5 ACOG regions, with region 4 representing the most respondents (31.0%) and region 3 representing the fewest 10.7% (Table 2). Most program directors came from academic programs (60.7%), with the remainder coming from community programs (34.5%) and military programs (4.8%). About 45% were in their current roles from 0 to 3 years with 19% reporting tenure of greater than 10 years.

**Table 2.** Program director demographics.

		Respondents	National
Type of program	Academic	51 (60.7)	118 (46.1)
	Community	29 (34.5)	131 (51.2)
	Military	4 (4.8)	7 (2.7)
Program size	<12 total residents	9 (10.7)	38 (14.8)
	12-28 total residents	57 (67.9)	194 (75.8)
	>28 total residents	18 (21.4)	24 (9.4)
Program region	Region 1 (Connecticut, Maine, Massachusetts, Newfoundland, New Hampshire, New York, Rhode Island, Vermont	17 (20.2)	52 (20.3)
	Region 2 (Delaware, Indiana, Kentucky, Michigan, New Jersey, Ohio, Pennsylvania)	17 (20.2)	60 (23.4)
	Region 3 (District of Columbia, Florida, Georgia, Maryland, North Carolina, South Carolina, Virginia, West Virginia)	9 (10.7)	42 (16.4)
	Region 4 (Alabama, Arkansas, Illinois, Iowa, Kansas, Louisiana, Manitoba, Minnesota, Mississippi, Missouri, Nebraska, Oklahoma, Tennessee, Texas, Wisconsin)	26 (31.0)	63 (24.6)
	Region 5 (Arizona, Armed Forces District, British Columbia, California, Colorado, Hawaii, Nevada, New Mexico, Oregon, Utah, Washington)	15 (17.9)	39 (15.2)
Years in role	0-3	34 (40.5)	NA
	3-5	10 (11.9)	NA
	5-10	24 (28.6)	NA
	>10	16 (19.0)	NA
Program uses milestones	Yes	80 (95.2)	NA
	No	4 (4.8)	NA

Abbreviation: NA, not applicable.

n = 84. Data are expressed as No. (%).

About 87% of residents were familiar with milestone-based feedback (Table 3). Most of the residents believed that milestone-based feedback is useful (62.7%) and fair (70.0%). Smaller majorities found them to be accurate (55.4%), timely (50.4%), and actionable (54.4%). Of note, however, 19.5% and 14.4% of respondents believed that it is not timely or actionable, respectively.

**Table 3.** Resident and program director perceptions of milestone-based feedback.

	Residents (n = 631)	Program directors (n = 84)	*P* value
Milestone-based feedback is useful			
Agree or strongly agree	361 (62.7)	62 (74.7)	.031
Disagree strongly disagree	67 (11.7)	13 (15.7)	
Milestone-based feedback is fair			
Agree or strongly agree	401 (70.0)	69 (83.1)	.012
Disagree strongly disagree	29 (5.1)	5 (6.0)	
Milestone-based feedback is accurate			
Agree or strongly agree	317 (55.4)	61 (73.5)	.002
Disagree strongly disagree	69 (12.1)	9 (10.8)	
Milestone-based feedback is timely			
Agree or strongly agree	289 (50.4)	47 (57.3)	.235
Disagree strongly disagree	112 (19.5)	14 (17.1)	
Milestone-based feedback is actionable			
Agree or strongly agree	311 (54.4)	49 (59.0)	.426
Disagree strongly disagree	82 (14.3)	15 (18.1)	
Milestone-based feedback captures surgical aptitude			
Agree or strongly agree	296 (51.8)	31 (37.8)	.016
Disagree strongly disagree	94 (16.4)	30 (36.5)	
Milestone-based feedback allows me to track how my/resident performance changes over time			
Agree or strongly agree	368 (64.3)	62 (74.7)	.059
Disagree strongly disagree	66 (11.5)	11 (13.3)	

Data are expressed as No. (%).

Numbers may not add up to sample size as respondents could be neutral or decline to answer.

Although 78.1% of program directors believed that milestone-based feedback is more administratively burdensome compared with traditional feedback systems, they had a more favorable perspective regarding the new system than residents. Of program directors surveyed, most believed that milestone-based feedback is useful (74.7%), fair (83.0%), accurate (76.5%), timely (57.3%), and actionable (59%).

In terms of tracking performance over time, 64.3% of residents and 74.7% of program directors believed that milestone-based feedback is an effective tool to track resident progression. However, 16.4% of residents and 36.6% of program directors believed that milestone-based feedback does not capture surgical aptitude and performance.

In all, 34 of 84 program directors (40.5%) and 92 of 631 residents (14.6%) who completed the survey left additional comments (see Table 4). Of these, 5 of 34 (14.7%) program directors and 4 of 92 (4.3%) residents felt positively about milestone-based feedback. Program director comments most often referenced the administrative burden that milestone-based feedback presented (47.1%). Other themes identified include that it is not comprehensive (26.5%) and that it is not actionable (23.5%). In terms of resident comments, the most referenced theme was that milestone-based feedback is not comprehensive enough and/or does not accurately evaluate residents (27.1%). Other themes include that it is too broad/not specific enough (21.7%), it is not actionable (21.7%), and it is not timely (18.4%).

**Table 4.** Themes identified in open-ended responses by domain queried.

Program directors
*Program directors felt positively about milestone-based feedback*
“We added milestones to our previous feedback system which has been an advantage but also time consuming and alone not enough”
“The milestones themselves are somewhat useful in directing the development of program curriculum and individual curriculum, but do not provide specific enough feedback to residents”
*Program directors felt milestone-based feedback was an Administrative Burden*
“The faculty found the milestones so cumbersome that they stopped giving written feedback and just used the time to fill out the milestones levels.”
“The problem is that despite my best effort, it [milestones] is now almost our entire focus for evaluation and feedback because we need to expend so much energy to meet the requirements for reporting every 6 months”
*Program directors felt milestone-based feedback was not comprehensive*
“Do not provide specific enough feedback to residents”
“Discourages written comments which is really what the residents find value in”
*Program directors felt milestone-based feedback provided not actionable feedback*
“They are more evaluative than meant to provide feedback”
“At the current time I am using both Milestones as well as the previous method of feedback because Milestones doesn’t as effectively capture the overall picture of the resident as a physician”
Residents
*Residents felt milestone-based feedback was helpful*
“The idea is good, but I still feel a significant lack of real time feedback”
“I think that milestone based feedback is better in that it targets specific areas. The caveat to this is that there is an action plan supported by the program in order to correct deficiencies, such as more structured surgical training, etc”
*Residents felt milestone-based feedback was too broad/not specific enough*
“It’s vague and not specific, does not allow for constructive feedback”
“I felt that my milestone evaluation report was generic and arbitrary. I think it wastes time during an evaluation where the program director could instead be giving you more specific and personal feedback”
*Residents felt milestone-based feedback was not comprehensive enough and/or did not accurately evaluate residents*
“Specific examples are the only way most of us are able to improve our weaknesses. There are too many checkboxes to click on our milestone feedback, and many residents feel that attendings hardly read the question before clicking an answer”
“Evaluation is often very standardized- i.e. an evaluator will check the standard milestone levels that are appropriate for PGY year rather than offering individual suggestions for improvement. Individual feedback on specifics is more useful”
*Residents felt milestone-based feedback was not actionable*
“Feedback provided on scales is generic and often reflects the relationship between the reviewer and the reviewed more than any actual thoughtful, actionable criticism”
“At this time we don’t know how to interpret the milestones evaluations or what to do with the information therefore it has not been extremely helpful in reviewing our performance”
*Residents felt milestone-based feedback was not timely enough*
“The Milestone feedback is extensive which means that it takes a long time for evaluators to complete the feedback and yet the result is not any better”
“We only receive this information twice per year, and for it to be meaningful and for us to make timely changes or improvements”

## Discussion

Our study demonstrates that milestone-based feedback has been widely integrated into Obstetrics and Gynecology residency programs nationwide. As seen in Table 3, most of the residents and program directors feel positively about milestone-based feedback and 64.3% of residents and 74.7% of program directors stated that milestone-based feedback is an effective tool to track resident performance over time. Nonetheless, our study identified issues of evaluation of surgical aptitude, administrative burden, timeliness, and ability to act on milestone-based feedback. Some of these issues were elucidated further by comments from both resident and program director respondents.

Only half of the residents and about a third of program directors believed that milestone-based feedback captures surgical aptitude and performance appropriately, suggesting that an additional system is needed to document proficiency in these areas. In 2013, the Johns Hopkins University Plastic Surgery residency program implemented the Comprehensive Observations of Resident Evolution (CORE) program in an effort to capture resident-specific operative strengths and weaknesses. They found that this tool increases immediate attending/trainee feedback and assessment transparency, enables trainee self-monitoring, and informs end-of-rotation reviews, program-wide assessments, and tailoring of training to address specific needs.^12^ Integrating milestones with innovative programs such as CORE may help improve the surgical aspect of resident evaluations.

As with previous studies, our study demonstrates that residency program directors feel that milestone evaluation and reporting has increased administrative burden.^5–7^ This is in direct contrast to the stated goals of the NAS. In our study, 75% of program directors reported that milestone-based feedback is more time-consuming than prior feedback systems. This could in part represent an early time investment in setting up evaluations to capture information for milestone assignment, but further evaluation over time is necessary to understand whether this administrative burden persists even when systems are in place.

Other concerns raised by this study include that milestone-based feedback is not actionable, as milestones are “more evaluative than meant to provide feedback.” Both residents and program directors noted that when residents are not performing at the expected level, there are not enough specific details on the shortcomings; hence, it is challenging to define a plan to address deficiencies. Furthermore, 20% of residents felt that milestone-based feedback was not timely and that it should be given at more frequent intervals for residents to be able to make timely changes and improvements. Comments from program directors highlight that spontaneous faculty comments have decreased on evaluation forms due to the increased time necessary to gather milestone-oriented information. This sentiment is highlighted several times in the resident comments as well. In addition, although milestone-based feedback is not meant to replace informal or verbal feedback, comments suggest that the administrative burden associated with milestone-based evaluation may also have taken away from real-time feedback. As the milestones are further developed, focusing on both streamlining the feedback submission process as to capture specific feedback in a timely manner without excessively burdening the submitter, as well as making sure that there are actionable plans to correct deficiencies once these are identified, may help to improve the acceptability and applicability of this system.

There are several limitations to our study. First, as with any survey study, there is a possibility of bias, as program directors and residents who chose to respond to the survey may differ from those who did not respond. We were unable to calculate an exact resident response rate given our inability to determine how many residents received the survey, although 12.6% of eligible residents did respond. Nonetheless, we believe that with greater than 600 residents who are well distributed by PGY class, region, and program size, we could sample a reasonable range of opinions. Similarly, there is potential for sampling bias among program directors, as evidenced by the fact that a slightly higher percentage of program directors in our sample work at academic hospitals than the national average. Furthermore, 40% of program directors have only been in their role for 0 to 3, so they may not have had much experience with the traditional milestone system as program directors. In addition, this study is confined to Obstetrics and Gynecology and has some shared findings with previous studies in other specialties but likely some results may be specific to Obstetrics and Gynecology as there are different milestones for each specialty. We also recognize that for our free response portion, it is likely those who feel strongly positively or negatively that will likely comment. Hence, we expected and received polarized answers. Finally, “milestone-based feedback” was not specifically defined on the survey which could lead to misunderstanding of this term by the respondents.

To our knowledge, our study is the first to assess residents and program director’s perceptions of milestone-based feedback in Obstetrics and Gynecology. Our study highlights the need for programs to continue to develop tools that capture and deliver milestone-based feedback more effectively with the goal of enhancing resident development. In addition, because milestones do not encompass the totality of the breadth of training needed to be a competent Obstetrician/Gynecologist, continuing other forms of evaluation and feedback is essential. Although this is certainly a challenging task, we believe that our findings will help guide program directors and residencies to further develop feedback tools that work optimally for residents and faculty in both surgical and nonsurgical specialties. As each specialty determines the optimal methods for measuring milestones, it is crucial to continue to evaluate and understand how residents and program directors experience milestone implementation.
